# Evaluation of eight lateral flow tests for the detection of anti-SARS-CoV-2 antibodies in a vaccinated population

**DOI:** 10.1186/s12879-023-08033-1

**Published:** 2023-02-23

**Authors:** Caitlin Greenland-Bews, Rachel L. Byrne, Sophie I. Owen, Rachel L. Watkins, Daisy Bengey, Kate Buist, Karina Clerkin, Camille Escadafal, Lorna S. Finch, Susan Gould, Emanuele Giorgi, Andy Hodgkinson, Larysa Mashenko, Darren Powell, Helen R. Savage, Caitlin R. Thompson, Lance Turtle, Jahanara Wardale, Dominic Wooding, Thomas Edwards, Ana Cubas Atienzar, Emily R. Adams

**Affiliations:** 1 Liverpool School of Tropical Medicine, Centre for Drugs and Diagnostics, Liverpool, UK; 2 FIND, Foundation for Innovative New Diagnostics, Geneva, Switzerland; 3 Department of Clinical Infection, Microbiology and Immunology, University of Liverpool, Liverpool, UK; 4 Global Access Diagnostics (GADx), Bedfordshire, UK; 5 Lancaster Medical School, Lancaster University, Lancaster, UK; 6 Biochemistry Department, Alder Hey Children’s Hospital, Liverpool, UK

**Keywords:** Antibody, Diagnostics, COVID-19, Vaccination, Serology

## Abstract

**Background:**

Rapid determination of an individual’s antibody status can be beneficial in understanding an individual’s immune response to SARS-CoV-2 and for initiation of therapies that are only deemed effective in sero-negative individuals. Antibody lateral flow tests (LFTs) have potential to address this need as a rapid, point of care test.

**Methods:**

Here we present a proof-of-concept evaluation of eight LFT brands using sera from 95 vaccinated individuals to determine sensitivity for detecting vaccination generated antibodies. Samples were analysed on eight different brands of antibody LFT and an automated chemiluminescent microparticle immunoassay (CMIA) that identifies anti-spike antibodies which was used as our reference standard.

**Results:**

All 95 (100%) participants tested positive for anti-spike antibodies by the chemiluminescent microparticle immunoassay (CMIA) reference standard post-dose two of their SARS-CoV-2 vaccine: BNT162b2 (Pfizer/BioNTech, n = 60), AZD1222 (AstraZeneca, n = 31), mRNA-1273 (Moderna, n = 2) and Undeclared Vaccine Brand (n = 2). Sensitivity increased from dose one to dose two in six out of eight LFTs with three tests achieving 100% sensitivity at dose two in detecting anti-spike antibodies.

**Conclusions:**

These tests are demonstrated to be highly sensitive to detect raised antibody levels in vaccinated individuals. RDTs are low cost and rapid alternatives to ELISA based systems.

**Supplementary Information:**

The online version contains supplementary material available at 10.1186/s12879-023-08033-1.

## Background

In the ongoing COVID-19 pandemic, the rapid development and emergency use authorisation (EUA) of multiple COVID-19 vaccines [1–3] within the first year of the SARS-CoV-2 pandemic was an unprecedented achievement. Large-scale national vaccination programmes including booster shots are widespread in high income countries [4, 5]. This sparked global discussion regarding vaccine equity [6, 7] and the large disparity in the accessibility of COVID-19 vaccines between high- and low- income countries, further vaccine hesitancy means that large populations remain unvaccinated. The use of monoclonal antibody therapies (mAbs) e.g., casirivimab/imdevimab for treatment of COVID-19 patients requires that patients are seronegative to be eligible for therapy and therefore require rapid determination of antibody status before treatment can begin [8]. Clinical trials have found that the combination only reduces mortality in patients who were seronegative [9].

Determination of antibody titres to a specific pathogen is commonly achieved through enzyme linked immunosorbent assay (ELISA), which are relatively accessible in high income countries, but less accessible in low- and middle- income countries (LMICs) [10]. Lateral flow tests (LFTs) however are a quick, point of care test that require minimal prior training that could be scaled up for population wide screening for presence of anti-SARS-CoV-2 antibodies. Since the COVID-19 pandemic, an enormous number of manufacturers have developed LFTs which have entered the market without standardisation, although the National Institute for Biological Standards and Control (NIBSC) now have available standards for anti-SARS-CoV-2 immunoglobulin. Still, there is minimal validation procedure for these tests, and to date the available data on these tests indicates variable performance [11–16]. Lack of consistent methodology and reference standards make comparison of results between these studies difficult. Currently the World Health Organization (WHO) only recommends the use of these tests in research settings and states that more data are required on LFT performance to determine their suitability as a tool in the COVID-19 pandemic and global vaccination programme [17].

An evaluation of multiple brands of antibody LFTs is required in vaccinated individuals at multiple time points to accurately assess their performance compared to a sensitive reference standard. To this aim we have conducted a laboratory evaluation of eight commercially available LFTs utilising stored serum samples with comparisons to an automated chemiluminescent microparticle immunoassay (CMIA) as a reference standard that is routinely used in clinical settings.

## Methods

### Study design and ethics

The NHS Research Ethics Committee (REC, UK) [REC reference:16/NW/0170] and the central Liverpool research ethics committee [Protocol Number: UoL001207] granted ethical approval for this work. The Integrated Research Application System (IRAS) Project ID is: 202413.

Participants were recruited from the Liverpool School of Tropical Medicine and University of Liverpool staff networks as well as members of the public through social media outreach. All participants were recruited from the Liverpool, Merseyside region of the UK. Participants were recruited onto an existing study (The Human Immune Responses to Acute Viral Infections study (AVIS), 16/NW/0160). Participants were recruited between January-December 2021. All participants gave written informed consent. Healthy individuals who had received or were due to receive their COVID-19 vaccination and were aged 18 years or over were recruited to the study. Individuals taking part in COVID vaccine trials were excluded from the study. Case record form (CRF) was completed by a trained member of staff to confirm eligibility. Participants were asked to provide a blood sample at days 21 (± 7 days), 42 (± 7 days) post dose one and two of their COVID-19 vaccine.

### Sample collection and processing

Venous blood (5 ml, plain serum tube) samples were collected by trained health care workers and processed on the same day of collection. Briefly, venous blood samples were centrifuged at 1500 g for 10 min and serum was aliquoted and stored at − 20 °C until testing. Following processing samples were stored at − 20 °C before testing in bulk.

### Lateral flow tests

LFTs were performed according to the instructions for use (IFU). Serum was allowed to thaw at room temperature for 15 min and vortexed for 5 s. A sample from each individual was tested on all brands of LFTs. According to individual IFU’s, 10–20 µl of serum was added to the sample well and 2–3 drops of manufacturer specified buffer solution was added. Tests were run for 10–15 min, according to IFU, and read independently by two readers. Where there was a disagreement a third reader was used. Failed tests were repeated once. Characteristics of the tests used are shown in Table 1. When no details on the antigen composition were provided in the IFU, the company was approached for further information. Although all tests used in this study detect both IgM and IgG antibodies, IgG was the focus of this investigation and results from IgM are not included. This is due to the longevity of IgG antibodies compared to IgM likely making them a more reliable target as proxy for immunity.

**Table 1:** Summary of Lateral Flow Test details including antigen, sample requirements and running time

LFT brand	Antigen used	Sample volume (µl)	Buffer volume (µl or drops)	Time to read (mins)
WANTAI SARS-CoV-2 Ab Rapid Test (Beijing Wantai Biological Pharmacy) (Wantai)	Spike-RBD	10	2 drops	15
Onsite COVID-19 IgG/IgM Rapid Test (CTK Biotech)) (CTK)	Spike	10	2 drops	15
COVID-19 Total Ab Device (Fortress Diagnostics LTd) (Fortress)	Spike- RBD	10	2 drops	10–15
NowCheck COVID-19 IgM/IgG Test (Bionote Co., LTD.) (Bionote)	Nucleoprotein	20	3 drops	10–15
Edinburgh Genetics COVID-19 Colloidal Gold Immunoassay Testing Kit, IgG/IgM Combined (Edinburgh Genetics)	Nucleoprotein	20	60 µl	10–15
Diagnostic Kit for SARS-CoV-2 IgM/IgG Antibody (Colloidal Gold) (Shanghai Kehua Bio-Engineering Co., Ltd.) (KHB)	Nucleoprotein	10	3 drops	15
SARS-CoV-2 IgM/IgG Ab Rapid Test (Qingdao HIGHTOP Biotech Co., Ltd.) (Qingdao)	Nucleoprotein and spike	10	2 drops	15–20
P4DETECT COVID-19 IgM/IgG (PRIME4DIA Co., Ltd) (Prime4Dia)	Nucleoprotein and spike	10	3 drops	10–15

*RBD* receptor binding domain

### Immunoassays

Samples were analysed by quantitative and semi-qualitative chemiluminescent microparticle immunoassay (CMIA) on the fully automated Alinity i system (Abbot, United States) as a reference standard. SARS-CoV-2 IgG II CMIA (Abbott, Ireland) was used to quantify anti-S-RBD IgG antibodies in serum samples. To distinguish between antibodies produced from natural infection from SARS-CoV-2 to those produced from vaccination the samples were also analysed using the SARS-CoV-2 IgG I assay (Abbott, Ireland), a semi-qualitative CMIA to detect anti-nucleocapsid IgG antibodies, a method that was utilised by Narasimhan et al*.* and found to be effective [18]. When using these assays, individuals positive for anti-nucleocapsid IgG antibodies are considered to have been naturally infected with SARS-CoV-2 and will also test positive for anti-S-RBD IgG antibodies. If an individual gives a negative test result for anti-nucleocapsid IgG antibodies but a positive result for anti-S-RBD IgG antibodies, then this individual is assumed to have not had a natural infection and has antibodies generated in response to vaccination. Following manufacturer recommendations, results higher or equal to 50 AU/ml when using the SARS-CoV-2 IgG II Quant assay were considered positive for anti-S-RBD IgG antibodies. Similarly, results higher than or equal to 1.4 S/C (Sample control index) when using the SARS-CoV-2 IgG I Qualitative assay were considered positive for anti-nucleocapsid IgG antibodies as per manufacturer’s instructions.

### Sensitivity calculation:

Sensitivity of the eight brands of AbLFT was calculated in reference to the results from the SARS-CoV-2 IgG II CMIA that quantifies anti-S-RBD IgG antibodies. Only this test was used as a reference standard as we wanted to assess the sensitivity of detecting those with antibodies from the vaccine which would be anti-spike given the main available vaccines during sample collection utilised the spike protein in their design. Sensitivity was calculated as a proportion of the number of positive and negative AbLFT results that were confirmed by CMIA. The results of which can be found in Additional file 1: Table S3.

### Model formulation

A binomial mixed effect model was designed to provide point-estimates for sensitivity based on the data collected from the laboratory evaluation. Due to the small sample size, binomial mixed models allowed us to borrow strength information across all individuals and estimate the sensitivity of each test more reliably than the conventional approach based on simple proportions. The model also analyses the impact of the key variables on the sensitivity of the different LFTs and to determine parameters for the calculation of the sensitivities and confidence intervals of each LFT at each dose. Details of modelling methods can be found in Additional file 1.

### Statistical analysis

We used binomial mixed models to account for the clustering arising from the administration of multiple tests on the same individuals. These models were used to assess the effect of the LFT brand and other factors on the risk of a positive test. More details on the binomial mixed models and a comparison with the standard approach for estimation of LFT sensitivity can be found in the Additional file 1. The statistical analysis was conducted in RStudio (Version: 2021.9.1.372).

## Results

A total of 95 participants were recruited and provided at least one blood sample post dose one or two. A total of 89 participants provided a sample post dose one and 69 provided a sample post dose two with 63 participants providing a sample after both dose one and two. Of the 95 participants, 63 (66.3%) were female with a mean age of 39 years. CMIA analysis was conducted on all samples and showed that seven (10.1%) individuals tested positive for anti-nucleoprotein antibodies post-dose one and six (8.7%) tested positive post-dose two. The decrease in positivity is due to an individual not providing a sample post-dose 2 rather than loss of anti-nucleoprotein antibodies between doses. Of the seven participants that tested positive, five had previously reported a positive PCR test prior to enrolment with the study. CMIA also found 88 (98.8%) samples tested positive for anti-S-RBD antibodies post dose 1 and 69 (100%) post dose 2.

### Sensitivities

Point estimates of sensitivity from the binomial mixed effect model and the standard percentage calculation were largely comparable and results from both are summarised in Tables 2 and 3. Sensitivity from the binomial mixed effect model for dose 1 ranged from 4.38% [CI95% 1.24, 8.40] for KHB to 95.43% [87.42, 97.42] for Fortress. For dose 2, sensitivities ranged from 20.15% [13.15, 30.02] for KHB to 99.30% [96.46, 99.73] for Fortress. Similarly, the standard percentage calculation for dose 1 ranged from 14.77% [CI95% 8.11, 23.94] for KHB to 97.72 [CI95% 92.3, 99.72] for Fortress. For dose 2, sensitivities ranged from 11.59% [CI95% 5.14, 21.57] for KHB to 100% [CI95% 94.79, 100] for CTK, Fortress and Bionote.

**Table 2:** Point estimates and 95% confidence intervals from the lateral flow test (LFT) sensitivity obtained from the fitted Binomial mixed model against proportional sensitivity, for each brand at Dose 1

LFT brand	Antigen	Model sensitivity (%) [CI95%]	Proportional sensitivity (%) [CI95%]
WANTAI SARS-CoV-2 Ab Rapid Test (Beijing Wantai Biological Pharmacy)	Spike-RBD	47.16 [36.79,58.20]	40.91 [30.54,51.91]
Onsite COVID-19 IgG/IgM Rapid Test (CTK Biotech)	Spike	86.58 [79.78,93.65]	88.64 [80.09, 94.41]
COVID-19 Total Ab Device (Fortress Diagnostics LTd)	Spike-RBD	95.43 [87.42,97.42]	97.72 [92.3, 99.72]
NowCheck COVID-19 IgM/IgG Test (Bionote Co., LTD.)	Nucleoprotein	76.24 [67.29, 85.08]	76.14 [65.86, 84.58]
Edinburgh Genetics COVID-19 Colloidal Gold Immunoassay Testing Kit, IgG/IgM Combined (Edinburgh Genetics)	Nucleoprotein	68.50 [58.01, 76.88]	69.32 [58.58, 78.71]
Diagnostic Kit for SARS-CoV-2 IgM/IgG Antibody (Colloidal Gold) (Shanghai Kehua Bio-Engineering Co., Ltd.)	Nucleoprotein	4.38 [1.24, 8.40]	14.77 [8.11, 23.94]
SARS-CoV-2 IgM/IgG Ab Rapid Test (Qingdao HIGHTOP Biotech Co., Ltd.)	Nucleoprotein and spike	69.33 [58.71, 78.19]	70.45 [59.78, 79.71]
P4DETECT COVID-19 IgM/IgG (PRIME4DIA Co., Ltd)	Nucleoprotein and spike	45.05 [34.13, 54.97]	37.50 [27.40, 48.47]

**Table 3:** Validation of point estimates and 95% confidence intervals from the lateral flow test (LFT) sensitivity obtained from the fitted Binomial mixed model against proportional sensitivity, for each brand at Dose 2

LFT brand	Nucleoprotein and Spike	Model sensitivity [CI95%]	Proportional sensitivity [CI95%]
WANTAI SARS-CoV-2 Ab Rapid Test (Beijing Wantai Biological Pharmacy)	Spike-RBD	76.58 [66.19, 82.18]	89.86 [80.21, 95.82]
Onsite COVID-19 IgG/IgM Rapid Test (CTK Biotech)	Spike	97.03 [92.46, 98.51]	100 [94.79, 100.00]
COVID-19 Total Ab Device (Fortress Diagnostics LTd)	Spike-RBD	99.30 [96.46, 99.73]	100 [94.79, 100.00]
NowCheck COVID-19 IgM/IgG Test (Bionote Co., LTD.)	Nucleoprotein	93.30 [86.68, 96.32]	100 [94.79, 100.00]
Edinburgh Genetics COVID-19 Colloidal Gold Immunoassay Testing Kit, IgG/IgM Combined (Edinburgh Genetics)	Nucleoprotein	89.79 [81.93, 93.24]	94.2 [85.82, 98.4]
Diagnostic Kit for SARS-CoV-2 IgM/IgG Antibody (Colloidal Gold) (Shanghai Kehua Bio-Engineering Co., Ltd.)	Nucleoprotein	20.15 [13.15, 30.02]	11.59 [5.14, 21.57]
SARS-CoV-2 IgM/IgG Ab Rapid Test (Qingdao HIGHTOP Biotech Co., Ltd.)	Nucleoprotein and spike	90.19 [82.70, 93.87]	95 [87.82, 99.09]
P4DETECT COVID-19 IgM/IgG (PRIME4DIA Co., Ltd)	Nucleoprotein and spike	74.95 [66.93, 84.41]	88.41 [78.43, 94.86]

Both the mixed effect model and the standard percentage calculation showed that six out of eight LFTs had a statistically significant increase in sensitivity estimates from dose 1 to dose 2. This may be indicative of higher antibody titres following a second vaccine dose making it easier to detect antibodies on LFT. Fortress did not have a statistically significant increase in sensitivity however had already achieved the highest sensitivity of all eight brands at dose 1.

Sensitivities of the tests when focussing on target antigen was varied. Three tests used nucleoprotein, two used both nucleoprotein and spike and three used spike alone, with two specifying the receptor binding domain (RBD) (Table 1). The tests that achieved the highest sensitivities post dose 2, CTK, Fortress and Bionote, all used different antigens of spike, spike-RBD and nucleoprotein respectively. The LFT with the lowest sensitivity was KHB which used nucleoprotein antigen.

### Impact of variables on test result (mixed effect model analysis)

Our mixed effect model found that vaccine brand and day of sampling (Day 21 vs day 42) had no significant effect on overall test result and were therefore removed from the model analysis (Additional file 1: Table S1). Dose had a significant, positive effect on positivity rate with more positive results being detected after dose 2 compared to dose 1 (Additional file 1: Table S2).

## Discussion

In this study we evaluated eight LFTs using sera from 95 vaccinated individuals, post-dose 1 and 2, to determine their sensitivity in detecting IgG antibodies specific to SARS-CoV-2 spike-RBD. We detected large variability in the sensitivities of these tests at different timepoints with Fortress having the highest sensitivity out of the eight tests evaluated, although specificity has not been considered in this study.

Overall, these results show LFTs can detect anti-S-RBD antibodies in vaccinated individuals and sensitivity increased with post-dose 2 samples. Sensitivity varies across the different brands and different antigens used with KHB demonstrating significantly lower sensitivity after both vaccine doses compared to the other 7 brands. Fortress showed the highest sensitivity in our vaccinated cohort and has also shown high sensitivity and specificity in other studies evaluating infected individuals [12, 19, 20] and has been utilised in a large-scale seroprevalence study in the UK [12, 19]. The variable sensitivities shown here and in other LFT evaluations [20, 21] highlights the importance of clinical evaluations to not only establish a potential use-case but to determine which brands are best suited for further implementation. Specificity was not calculated as part of this study however specificity of these brands has been evaluated using RT-PCR negative samples and pre-pandemic samples in previous work [20, 21]. Across these studies specificity ranged from 98.7 to 100% for the brands tested in this study. It was difficult to determine the impact the difference in antigen (spike and nucleoprotein) in each test had on sensitivity and more information on the antigens from each brand would be beneficial for future evaluations.

Binomial mixed model analysis found that the test results were not significantly impacted by day of sample collection (Day 21 or 42 post vaccine dose) which is consistent with findings indicating IgG antibodies are detectable between 21 and 60 days after vaccination [22, 23]. Similarly, vaccine brand did not significantly impact test results, both these findings highlight that wider testing could be flexible without compromising sensitivity.

Future work should include correlation studies to determine if a positive antibody LFT result is conducive to neutralising capacity in vaccinated individuals and if there is correlation between LFT line strength and protective antibody response. A recent review found imperfect correlation between presence of IgG and neutralising antibodies [24] however more investigation is required in this area.

Limitations of this work include the small sample size; we have tried to address this by the addition of a binomial model to improve reliability of our sensitivity calculations. Furthermore, although AbLFTs require less training, are cheaper and therefore are arguably a more accessible tool for assessing immunity of individuals. It should be acknowledged that these tests are also designed to be used with fingerpick samples which improves the accessibility compared to using serum, as we have here, which would require access to professionals capable of venepuncture and equipment for blood processing.

LFTs have the potential to be a valuable, point of care tool to aid in assessing antibody status and determine eligibility to life-saving monoclonal-antibody therapies. More generally, establishing the sensitivity of these AbLFTs in different populations provides useful data to guide their potential use-case moving forward. Our study has provided an evaluation of multiple brands of LFT in vaccinated people across multiple timepoints and the variation observed in our study and other evaluations [11–15] highlights the importance for robust evaluation methods and standardisation to be implemented.

## Supplementary Information

**Additional file 1. **Supplementary Materials.

## Data Availability

The datasets used and/or analysed during the current study will be made available from the corresponding author on reasonable request.

## Abbreviations

AbLFT: Antibody lateral flow test
CMIA: Chemiluminescent microparticle immunoassay
CRF: Case record form
ELISA: Enzyme linked immunosorbent assay
EUA: Emergency use authorisation
IFU: Instructions for use
LFT: Lateral flow test
LMIC: Lower Middle Income Country
NIBSC: National Institute for Biological Standards and Control
RBD: Receptor binding domain
WHO: World Health Organization
