# Supplementary material for: Evaluation of eight lateral flow tests for the detection of anti-SARS-CoV-2 antibodies in a vaccinated population
Source: BMC Infect Dis. 2023 Feb 23;23:110. doi: 10.1186/s12879-023-08033-1 (PMC9947870; doi:10.1186/s12879-023-08033-1)
Supplement: Supplementary file 1 — Additional file 1. Supplementary Materials. [file 12879_2023_8033_MOESM1_ESM.docx]

**Supplementary Materials:**

Let $Y_{ij}$denote the test outcome for the i-th individual (1=”positive”, 0=”negative”) using the j-th brand. We then assume that the$Y_{ij}$conditionally on a individual-level random effect, $Z_{i}$, follows mutually independent Bernoulli variables with probability $p_{ij}$for a positive test result (i.e. $Y_{ij}=1$), such that

$log\left\{ \frac{p_{ij}}{1-p_{ij}} \right\}=\alpha_{j}+{\beta d}_{ij}+Z_{i}$ [1]

where $\alpha_{j}$is a brand-specific intercept and $d_{ij}$is the number of the dose with associated regression coefficient $\beta$. Finally, we use $\sigma^{2}$to denote the variance of $Z_{i}$.

We carry out parameter estimation of the model in [1] using the Gaussian quadrature methods implemented in the glmer function of the lme4 (25) package in R.

We compute the sensitivity of a test as follows. Let $Y_{S}$denote the outcome test for the reference test, and $Y_{k}$the outcome of the test from any other brand. Let $P\left[ A | B \right]$ denote the probability of event A, given we have observed event B; the sensitivity of the k-th test, based on the model in [1], is then defined as

$P\left[ Y_{k}=1 | Y_{S}=1 \right]=\int_{-\infty}^{\infty} f(z |Y_{S}=1)P\left[ Y_{k}=1 | z \right]dz$ [2]

where $f(z |Y_{S}=1)$ is the density function of the individual-level random effects conditioned to having observed a positive reference test and $P\left[ Y_{k}=1 | z \right]$is the probability of a positive test based on the k-th brand, as defined in [1]. To compute the integral in [2] we use a quadrature approach to carry out numerical integration.

To compute the confidence intervals of the sensitivity estimate in [2], we proceed as follows.

1. We simulate 1000 samples for $\beta$and $\sigma^{2}$ from a Multivariate Gassian distribution with mean given by the maximum likelihood estimates of $\beta$and $\sigma^{2}$, and covariance matrix given by the inverse of the negative of the observed Fisher information.
2. For each of the 1000 samples simulated in the previous step, we compute the corresponding estimates of the sensitivity according to [2].
3. Using the simulated sensitivity values from the previous step, we compute the 0.025 and 0.975 quantiles to obtain a 95% confidence interval.

| **Table S1.** Maximum likelihood estimates of the model parameters in equation [1]. | | |
| --- | --- | --- |
| Parameter | Estimate | Confidence interval |
| $\alpha_{ij}$  CTK  EdGen  Fortress  Hightop  KHB  NowCheck  NPRef  P4D  SpikeRef  Wantai | 4.560  2.022  6.941  2.117  -6.559  2.962  -8.064  -0.419  8.815  -0.205 | (3.278, 5.842)  (0.990, 3.053)  (5.045, 8.837)  (1.079, 3.154)  (-8.007, -5.111)  (1.863, 4.061)  (-9.808, -6.319)  (-1.381, 0.544)  (5.722, 11.908)  (-1.168, 0.758) |
| $\beta$ | 3.169 | (2.483, 3.854) |
| $\sigma^{2}$ | 13.484 | (8.470, 21.686) |

**Validation of Binomial mixed model point estimates:**

Point estimates of LFT sensitivity from the binomial mixed model analysis were validated against proportional sensitivity that was calculated in Excel 2019 (Microsoft 365). Results from this validation are shown in Table S2 and S3.

| **Table S2:**  Point estimates and 95% confidence intervals from the lateral flow test (LFT) sensitivity obtained from the fitted binomial mixed model, for each brand at Dose 1 and 2. | | | |
| --- | --- | --- | --- |
| **LFT Brand** | **Antigen** | **Dose 1 Sensitivity (%) [CI95%]** | **Dose 2 Sensitivity (%) [CI95%]** |
| **WANTAI SARS-CoV-2 Ab Rapid Test (Beijing Wantai Biological Pharmacy)** | Spike-RBD | 47.16 [36.79,58.20] | 76.58  [66.19, 82.18] |
| **Onsite COVID-19 IgG/IgM Rapid Test (CTK Biotech)** | Spike | 86.58 [79.78,93.65] | 97.03  [92.46, 98.51] |
| **COVID-19 Total Ab Device (Fortress Diagnostics LTd)** | Spike-RBD | 95.43 [87.42,97.42] | 99.30  [96.46, 99.73] |
| **NowCheck COVID-19 IgM/IgG Test (Bionote Co., LTD.)** | Nucleoprotein | 76.24  [67.29, 85.08] | 93.30  [86.68, 96.32] |
| **Edinburgh Genetics COVID-19 Colloidal Gold Immunoassay Testing Kit, IgG/IgM Combined (Edinburgh Genetics)** | Nucleoprotein | 68.50  [58.01, 76.88] | 89.79  [81.93, 93.24] |
| **Diagnostic Kit for SARS-CoV-2 IgM/IgG Antibody (Colloidal Gold) (Shanghai Kehua Bio-Engineering Co., Ltd.)** | Nucleoprotein | 4.38  [1.24, 8.40] | 20.15  [13.15, 30.02] |
| **SARS-CoV-2 IgM/IgG Ab Rapid Test (Qingdao HIGHTOP Biotech Co., Ltd.)** | Nucleoprotein and Spike | 69.33  [58.71, 78.19] | 90.19  [82.70, 93.87] |
| **P4DETECT COVID-19 IgM/IgG (PRIME4DIA Co., Ltd)** | Nucleoprotein and Spike | 45.05  [34.13, 54.97] | 74.95  [66.93, 84.41] |

| **Table S3:** Numbers of positive and negative results from each brand of AbLFT and the gold standard SARS-CoV-2 II CMIA for samples collected at dose 1 and dose 2. Sensitivity was calculated using these results. | | | | |
| --- | --- | --- | --- | --- |
| **Dose 1** | | | | |
|  |  | **SARS-CoV-2 II CMIA** | | **Sensitivity (%), [95%]** |
| **Brand** | **AbLFT** | Positive | Negative |  |
| **Hightop** | Positive | 62 | 0 | 70.45 [59.78-79.71] |
|  | Negative | 26 | 1 |  |
| **Wantai** | Positive | 36 | 0 | 40.91 [30.54-51.91] |
|  | Negative | 52 | 1 |  |
| **CTK** | Positive | 78 | 0 | 88.64 [80.09-94.41] |
|  | Negative | 10 | 1 |  |
| **Fortress** | Positive | 86 | 0 | 97.72 [92.3-99.72] |
|  | Negative | 2 | 1 |  |
| **KHB** | Positive | 13 | 0 | 14.77 [8.11-23.94] |
|  | Negative | 75 | 1 |  |
| **P4D** | Positive | 33 | 0 | 37.50 [27.40-48.47] |
|  | Negative | 55 | 1 |  |
| **NwC** | Positive | 67 | 0 | 76.14 [65.86-84.58] |
|  | Negative | 21 | 1 |  |
| **EdGen** | Positive | 61 | 0 | 69.32 [58.58-78.71] |
|  | Negative | 27 | 1 |  |
| **Dose 2** | | | | |
|  |  | **SARS-CoV-2 II CMIA** | | **Sensitivity (%), [95%]** |
| **Brand** | **AbLFT** | Positive | Negative |  |
| **Hightop** | Positive | 66 | 0 | 95 [87.82-99.09] |
|  | Negative | 3 | 0 |  |
| **Wantai** | Positive | 62 | 0 | 89.86 [80.21-95.82] |
|  | Negative | 7 | 0 |  |
| **CTK** | Positive | 69 | 0 | 100 [94.79 - 100.00] |
|  | Negative | 0 | 0 |  |
| **Fortress** | Positive | 69 | 0 | 100 [94.79 - 100.00] |
|  | Negative | 0 | 0 |  |
| **KHB** | Positive | 8 | 0 | 11.59 [5.14-21.57] |
|  | Negative | 61 | 0 |  |
| **P4D** | Positive | 61 | 0 | 88.41 [78.43-94.86] |
|  | Negative | 8 | 0 |  |
| **NwC** | Positive | 69 | 0 | 100 [94.79 - 100.00] |
|  | Negative | 0 | 0 |  |
| **EdGen** | Positive | 65 | 0 | 94.2 [85.82-98.4] |
|  | Negative | 4 | 0 |  |
